# Effect of Novel Polymer-Free Nitrogen-Doped Titanium Dioxide Film–Coated Coronary Stent Loaded With Mycophenolic Acid

**DOI:** 10.3389/fbioe.2021.650408

**Published:** 2021-10-29

**Authors:** Jae Won Shim, Sung Soo Kim, Hyun Kuk Kim, In Ho Bae, Dae Sung Park, Jun-Kyu Park, Jae Un Kim, Han Byul Kim, Min Young Lee, Joong Sun Kim, Jung Ha Kim, Bon-Sang Koo, Kang-Jin Jeong, Sun-Uk Kim, Min Chul Kim, Doo Sun Sim, Young Joon Hong, Youngkeun Ahn, Kyung Seob Lim, Myung Ho Jeong

**Affiliations:** ^1^ Korea Cardiovascular Stent Research Institute, Jangsung, South Korea; ^2^ Cardiovascular Research Center, Chonnam National University Hospital, Gwangju, South Korea; ^3^ Division of Cardiology, Chosun University Hospital, Gwangju, South Korea; ^4^ Research Institute of Medical Sciences, Chonnam National University, Gwangju, South Korea; ^5^ CGBio. Co. Ltd., Jangsung, South Korea; ^6^ College of Pharmacy, Research Institute of Pharmaceutical Sciences, Kyungpook National University, Daegu, South Korea; ^7^ College of Veterinary Medicine, Chonnam National University, Gwangju, South Korea; ^8^ Futuristic Animal Resource and Research Center, National Primate Research Center, Korea Research Institute of Bioscience and Biotechnology, Chungbuk, South Korea

**Keywords:** stents, myocophenolic acid, coronary artery, titanium coating, percutaneous coronary intervention, restenosis, inflammation

## Abstract

**Background:** Titanium is commonly used in blood-exposed medical devices because it has superior blood compatibility. Mycophenolic acid inhibits the proliferation of vascular smooth muscle cells. This study examined the effect of a non-polymer TiO_2_ thin film–coated stent with mycophenolic acid in a porcine coronary overstretch restenosis model.

**Methods:** Thirty coronary arteries in 15 pigs were randomized into three groups in which the coronary arteries were treated with a TiO_2_ film–coated stent with mycophenolic acid (NTM, *n* = 10), everolimus-eluting stent with biodegradable polymer (EES, *n* = 10), or TiO_2_ film–coated stent (NT, *n* = 10). A histopathologic analysis was performed 28 days after the stenting.

**Results:** There were no significant intergroup differences in injury score, internal elastic lamina area, or inflammation score. Percent area stenosis was significantly smaller in the NTM and EES groups than in the NT group (36.1 ± 13.63% vs. 31.6 ± 7.74% vs. 45.5 ± 18.96%, respectively, *p* = 0.0003). Fibrin score was greater in the EES group than in the NTM and NT groups [2.0 (range, 2.0–2.0) vs. 1.0 (range, 1.0–1.75) vs. 1.0 (range, 1.0–1.0), respectively, *p <* 0.0001]. The in-stent occlusion rate measured by micro-computed tomography demonstrated similar percent area stenosis rates on histology analysis (36.1 ± 15.10% in NTM vs. 31.6 ± 8.89% in EES vs. 45.5 ± 17.26% in NT, *p* < 0.05).

**Conclusion:** The NTM more effectively reduced neointima proliferation than the NT. Moreover, the inhibitory effect of NTM on smooth muscle cell proliferation was not inferior to that of the polymer-based EES with lower fibrin deposition in this porcine coronary restenosis model.

## Introduction

The treatment of acute myocardial infarction (AMI) has rapidly evolved over the past few decades from thrombolysis therapy to coronary stents including bare metal stents (BMS), metal-based drug-eluting stents (DES), and bioresorbable vascular scaffolds (BVS). DES with durable polymers such as paclitaxel (Taxus®) and sirolimus (Cypher®) have successfully surmounted the high restenosis rate after percutaneous coronary balloon angioplasty (PTCA) and/or BMS implantation. However, first-generation DES reduced in-stent restenosis (ISR) rates compared to former treatments, delayed re-endothelialization and late stent thrombosis (LST) have emerged as major concerns (Stettler et al., 2007; Wenaweser et al., 2008). The permanent polymer coatings of BMS were related with poor re-endothelialization and LST (Murphy et al., 1992; Nebeker et al., 2006; Stone et al., 2007). To overcome this limitation, biocompatible and/or biodegradable polymers were applied in second- and third-generation DES (Meredith et al., 2005). As a result, stent thrombosis was significantly reduced compared to first-generation DES (Leon et al., 2010). Although considerably improved, polymers have several disadvantages such as chronic inflammation and impaired arterial healing of stented lesions (van der Giessen et al., 1996). Therefore, patients who underwent DES implantation were recommended much longer antiplatelet therapy than those treated with BMS.

N-TiO_2_ thin film has been used for the purpose of improving the biocompatibility with the advantage of preventing the release of metal ions and providing an environment favorable for secondary material adhesion. In addition, the N-TiO_2_ thin film is one of the coating materials in the spotlight of the implant material due to the inhibition of inflammation and sulfate effect.

We developed a non-polymer DES to solve the problems of the polymer-based DES. The titanium dioxide (TiO_2_) film coating method using a plasma-enhanced chemical vapor deposition (PECVD) technique was applied to fabricate polymer-free DES in our previous studies (Lim et al., 2014; Sim et al., 2016). Mycophenolic acid (MPA), an immunosuppressive agent with several -limus derivatives (biolimus, sirolimus, everolimus, tacrolimus, etc.), is the most commonly used coronary stent coating.

The aim of this study was to evaluate the possibility and efficacy of non-polymer TiO_2_ thin film–coated stent with MPA in a porcine coronary overstretch restenosis model.

## Methods

### Materials

MPA (6-[1,3-Dihydro-7-hydroxy-5-methoxy-4-methyl-1-oxoisobenzofuran-6-yl]-4-methyl-4-hexanoic acid) was used in this study. The MPA was purchased from Sigma-Aldrich Co. (St. Louis, MO, United States). Poly-l-lactide (PLLA; 0.80–1.2 dl/g of inherent viscosity in chloroform at 0.1 w/v% at 25°C) was purchased from EVONIK (United Kingdom). Everolimus was purchased from LC Laboratories (United States). All other reagents were of analytical grade.

### Fabrication of Everolimus-Eluting Stent With Biodegradable Polymer

Stent material was cobalt chromium (Co-Cr) alloy. The bare metal stent was fabricated using a laser cutting machine (Rofin, Starcut, Hamburg, Germany) according to Chonnam National University Hospital (CNUH) stent design (Lim et al., 2013). The stent was coated with everolimus (20 mg) and PLLA (20 mg) using an ultrasonic spray coating system according to a previously reported method (Lim et al., 2016).

### Preparation of TiO_2_ Film–Coated Stent With MPA

**FIGURE 1:**
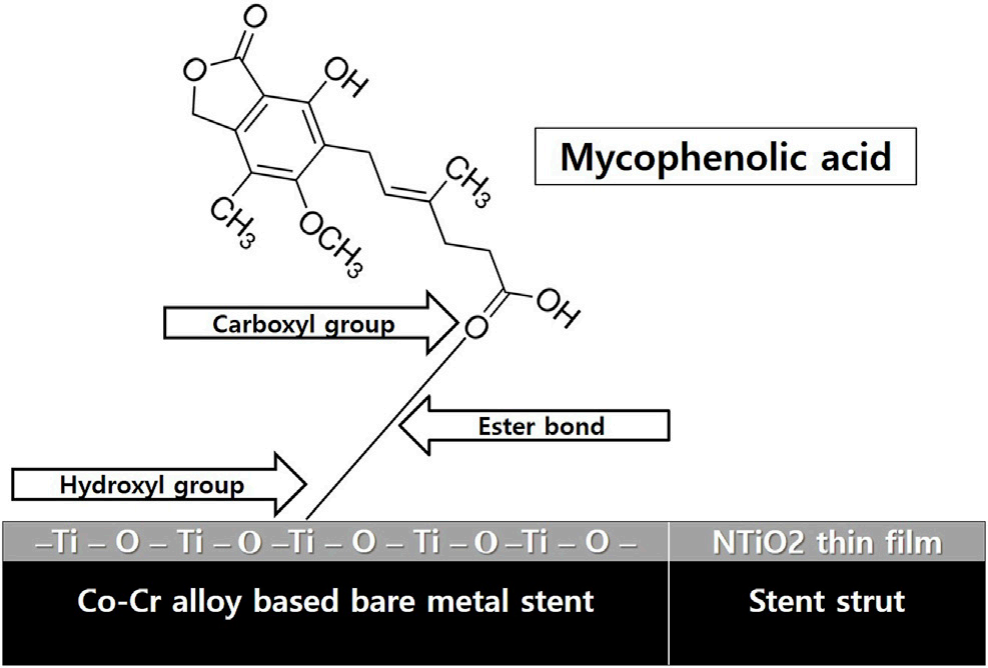
Schematic illustration of NTM. NTM, TiO_2_ film–coated stent with mycophenolic acid.

TiO_2_ thin film was deposited onto a CNUH BMS (3.0 × 16 mm) using the PECVD technique. MPA was coated without polymer according to a previously reported method (Song et al., 2009; Song et al., 2011; Lim et al., 2014). Briefly, the surface of the TiO_2_ thin film was modified using a water plasma method to introduce hydroxyl groups. To form an -OH functional group, it was reacted with high-purity Ar, O2, N2 gas at 4 sccm for 4 h at 400°C in a 50 mtorr vacuum through RF low-temperature plasma process. The MPA solution was subjected to an applied voltage of 20 kV, a radiation distance of 10 cm and 0.6 ml at room temperature. Electrospinning was then performed on top of the stent under a flow rate condition of 0.5 m/h. The MPA was then chemically grafted to the stent surface through the ester bonds between the carboxyl groups in the drugs and the hydroxyl groups of the modified TiO_2_ film (Figure 1). A mean 25 mg/ml of MPA was grafted onto the NTM. *In vitro* drug release kinetics were estimated using an ultraviolet–visible spectrophotometer as reported previously (Sim et al., 2016).

### *In vitro* Release Test of Everolimus or MPA Onto Coated Stent

The *in vitro* experiment of drug release was performed in a phosphate-buffered saline solution at 37°C. Further, the MPA or everolimus coated stent was placed at 37°C for 1, 4, 7, 14, 21, 30 d in the shaking incubator. The drug coated stents were placed in 50 ml of PBS, and at different time intervals, the sample solution was exchanged with fresh PBS of the same volume. The NaCl solution aliquots containing the released drug were injected into a Kromasil column 215 mm ID (Eka Chemicals AB, Separation Product, SE-445 80 Bohus, Sweden). The chromatographic conditions were as follows: column temperature 70°C; eluent water-acetonitrile (CH_3_CN); 5 min linear gradient 75–90% (v/v) CH_3_CN; 3 min 90% (v/v) CH_3_CN isocratic; 2 min linear gradient 90–75% (v/v) CH_3_CN; flow rate 1.0 ml/min; detection UV, 215 nm; calibrated measurement range 0.1–10.0 mg/L; and detection limit approx. 0.01 mg/L. In each case, the eluents, water, and CH_3_CN, contained 0.8 ml ortho-phosphoric acid (85%) perlites. The concentration of the drug was analyzed by HPLC according to the manufacturer’s protocol. The stent was inserted and consequently expanded in the silicone tubing using a balloon (3.0 × 16 mm) with pressure. Dipping the ends of the silicone tubing in 5 ml of PBS in a thermostatic water tank ensured that PBS was being circulated through the tubing. The concentration of drugs released was calculated by comparison with a drug standard curve, and it was expressed in a cumulative manner. The drug release rate of the stent was measured using a peristaltic pump (Jenie Well, Seoul, Korea).

### Stent Surface Evaluation and Chemical Elemental Analysis

The surface morphologies of the NTM and NT were investigated using scanning electron microscopy (SEM; TESCAN, MIRA3 LMU, Brno. Czech Republic). An energy-dispersive X-ray spectroscopy (EDS) test was performed to confirm the stent surface chemical characterization in NTM and NT. The topography analysis using atomic force microscopy (AFM) gives the hydrophilicity of TiO_2_ on the metal stent and the roughness of the bilayer film surface formed by MPA coating on it.

### Animal Preparation and Stent Implantation

The study animals were castrated male pigs weighing 20–25 kg. To prevent acute thrombosis after stenting, premedication with aspirin 100 mg and clopidogrel 75 mg per day was given for 5 days before the procedure. On the procedure day, the pigs were anesthetized with zolazepam and tiletamine (2.5 mg/kg, Zoletil50^®^, Virbac, Caros, France), xylazine (3 mg/kg, Rompun^®^, Bayer AG, Leverkusen, Germany), and azaperone (6 mg/kg, Stresnil^®^, Janssen-Cilag, Neuss, Germany). They received supplemental oxygen continuously through an oxygen mask. Subcutaneous 2% lidocaine was administered at the cut-down site, the left carotid artery was surgically exposed, and a 7-French sheath was inserted.

Continuous hemodynamic and surface electrocardiographic monitoring were performed throughout the procedure. Next, 5,000 units of heparin was administered intravenously as a bolus prior to the procedure, the target coronary artery was engaged using standard 7-French guide catheters, and control angiography of both coronary arteries was performed using a nonionic contrast agent in two orthogonal views.

The stent was deployed by inflating the balloon and the resulting stent-to-artery ratio was 1.3:1. Coronary angiograms were obtained immediately after stent implantation. Then, all equipment was removed and the carotid artery was ligated.

Four weeks after stenting, the animals underwent follow-up angiography in the same orthogonal views before being sacrificed by a 20-ml potassium chloride intracoronary injection.

The hearts were removed and the coronary arteries were pressure-perfusion fixed at 110 mmHg in 10% neutral buffered formalin overnight. Each stented artery was step-sectioned, processed routinely for light microscopy, and stained for histological analysis. All specimens were evaluated using micro-computed tomography (M-CT).

### Study Groups

The pigs were randomly divided into three groups: NTM (TiO_2_ film–coated stent with MPA, 3.0 × 16 mm, *n* = 10); EES (everolimus-eluting stent with biodegradable polymer, 3.0 × 16 mm, *n* = 10); and NT TiO_2_ film–coated stent, 3.0 × 16 mm, *n* = 10).

A total of 15 pigs were used in this study (15 pigs, 30 coronary arteries, 10 coronary arteries in each group). An NTM, EES, and NT were randomly implanted in the left anterior descending artery and left circumflex artery in each pig.

### Histopathological and M-CT Analyses

A histopathological evaluation of each artery was performed by an experienced cardiovascular pathologist. The specimens were embedded and sections 3–4-µm-thick were obtained approximately 1 mm apart and stained with hematoxylin-eosin and Carstairs’ for histological analysis. Measurements of the histopathologic sections were performed using a calibrated microscope, digital video imaging system, and microcomputer program (Visus 2000 Visual Image Analysis System, IMT Tech, CA, United States). Borders were manually traced for lumen area, area circumscribed by the internal elastic lamina, and the innermost border of the external elastic lamina (external elastic lamina area). A morphometric analysis of the neointimal area for a given vessel was calculated as the measured internal elastic lamina area minus the lumen area. The measurements were made on five cross-sections from the proximal and distal ends and the three midpoints of each stented segment. Histopathologic stenosis was calculated as 100 × [1–(lesion lumen area/lesion internal elastic lamina area)] (Schwartz et al., 1992).

The harvested stent specimen was stored in formaldehyde solution. A 1.5-ml Eppendorf tube was filled with clay, and the clay was turned with a V shape to hold the stent during contrast agent staining. The stents were taken from the solution and placed vertically in the V-shaped opening in the clay. Each stent had to be fixed with the clay such that there was no movement of the stent inside the Eppendorf tube. One milliliter of omnihexol, the contrast agent, was drawn up using a 5-ml syringe and injected through the opening at the center of the stent. The stent was incubated with contrast agent overnight and subjected to M-CT imaging (Che et al., 2012). All results were interpreted by two independent pathologists in a blinded fashion.

### Evaluation of Arterial Injury

The arterial injury at each strut site was determined by the anatomic structures penetrated by each strut. A numeric value was assigned as previously described by Schwartz et al. (1992): 0 = no injury; 1 = break in the internal elastic membrane; 2 = perforation of the media; and 3 = perforation of the external elastic membrane to the adventitia. The average injury score for each segment was calculated by dividing the sum of the injury scores by the total number of struts at the examined section.

### Evaluation of Inflammation Scores, Neointimal Reaction, and Fibrin Score

Inflammation of each individual strut was graded as follows: 0 = no inflammatory cells surrounding the strut; 1 = light, noncircumferential lymphohistiocytic infiltrate surrounding the strut; 2 = localized, moderate to dense cellular aggregate surrounding the strut non-circumferentially; and 3 = circumferential dense lymphohistiocytic cell infiltration of the strut. The inflammation score for each cross-section was calculated by dividing the sum of the individual inflammation scores by the total number of struts at the examined section (Schwartz et al., 2008). Ordinal data for fibrin were collected on each stent section using a scale of 0–3 as previously reported (Suzuki et al., 2001).

### Statistical Analysis

The statistical analysis was performed using SPSS version 15 (SPSS, Chicago, IL, United States). The data are presented as mean ± SD. Unpaired Student’s t test or analysis of variance was used to compare the stent groups. Ordinal measurements such as injury score, fibrin score, and inflammation score were analyzed using the Kruskal‒Wallis test. Non-parametric results are presented as median and interquartile range. Values of *p* < 0.05 were considered statistically significan.

## Results

### Stent Surface Evaluation of NTM and NT Using SEM

**FIGURE 2:**
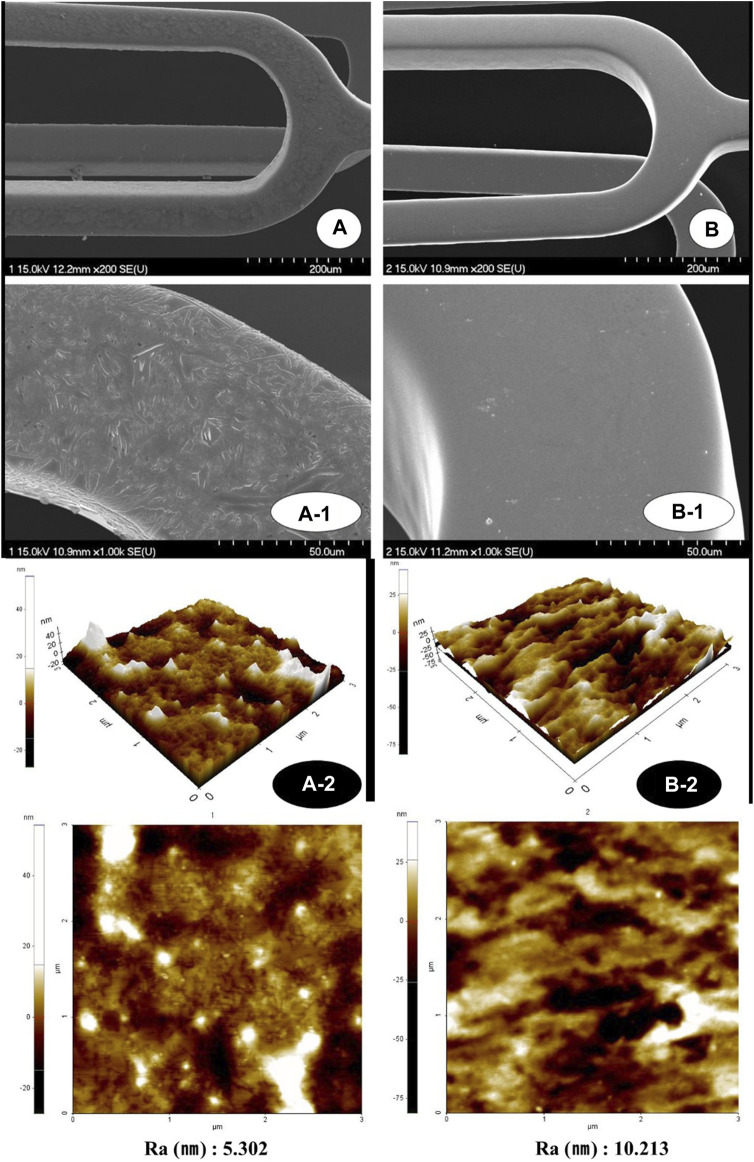
Surface morphology images of NTM [**(A)**; ×200, A-1; ×1,000, A-2; 3 × 3 µm] and NT using AFM and scanning electron microscopy [**(B)**; ×200, B-1; ×1,000, B-2; 3 × 3 µm]. AFM, Atomic force microscopy; NTM, TiO2 film–coated stent with mycophenolic acid; NT, TiO_2_ film–coated stent.

Figure 2 shows SEM images of the NTM and NT with the MPA or titanium thin film coating. The stent coated surface of the NTM and NT stents is quite uniform and smooth. While the surface of untreated NT does not show anything particular, the modified NTM shows wavelike structure at the edge of the foam. This wavelike structure is expected to come from the result of after coating drying. The modified NTM surface was observed to smooth out due to TiO_2_ grafting.

### AFM Analysis

The average surface roughness of each was analyzed as A-2 5.3 nm and B-2 10.2 nm (Figure 2). Overall uniformity is shown without significant change. The three-dimensional shape shows a dramatic decrease in root mean square (RMS) in the MPA group compared to the MPA untreated group. In contrast, the RMS roughness on the surface of the MPA treatment group was reduced. This indicates that the bonding force between the TiO_2_ thin film and the MPA coating material is excellent.

### EDS Analysis

**FIGURE 3:**
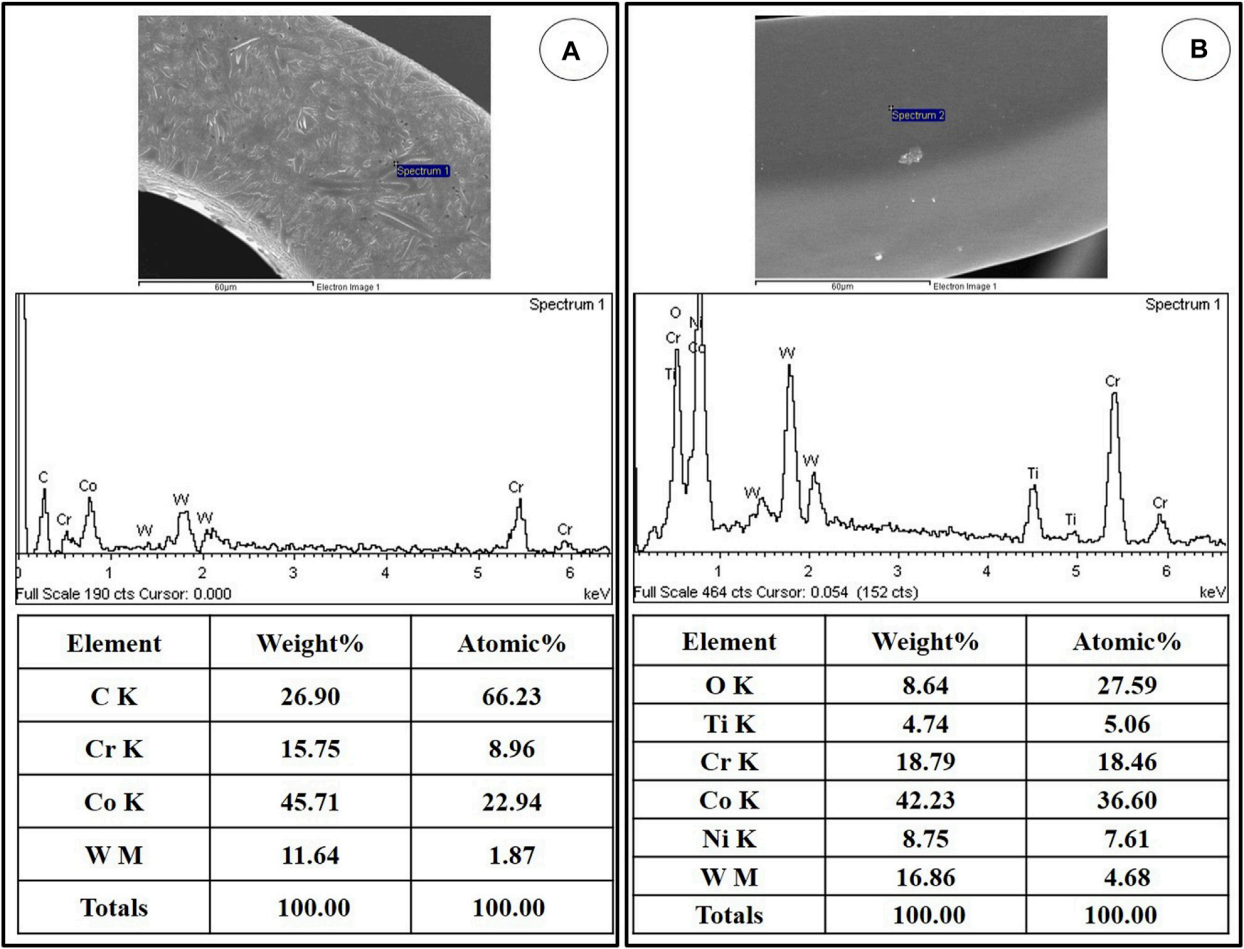
Representative EDS spectra showing the surface composition of NTM **(A)** and NT **(B)**. EDS, Energy-dispersive X-ray spectroscopy; NTM, TiO_2_ film–coated stent with mycophenolic acid; NT, TiO_2_ film–coated stent.

The qualitative EDS analysis confirmed the titanium coating (Figure 3B) and the titanium-coated stent surface covered with MPA (Figure 3A).

### *In vitro* Release Kinetics Analysis of EES or NTM

**FIGURE 4:**
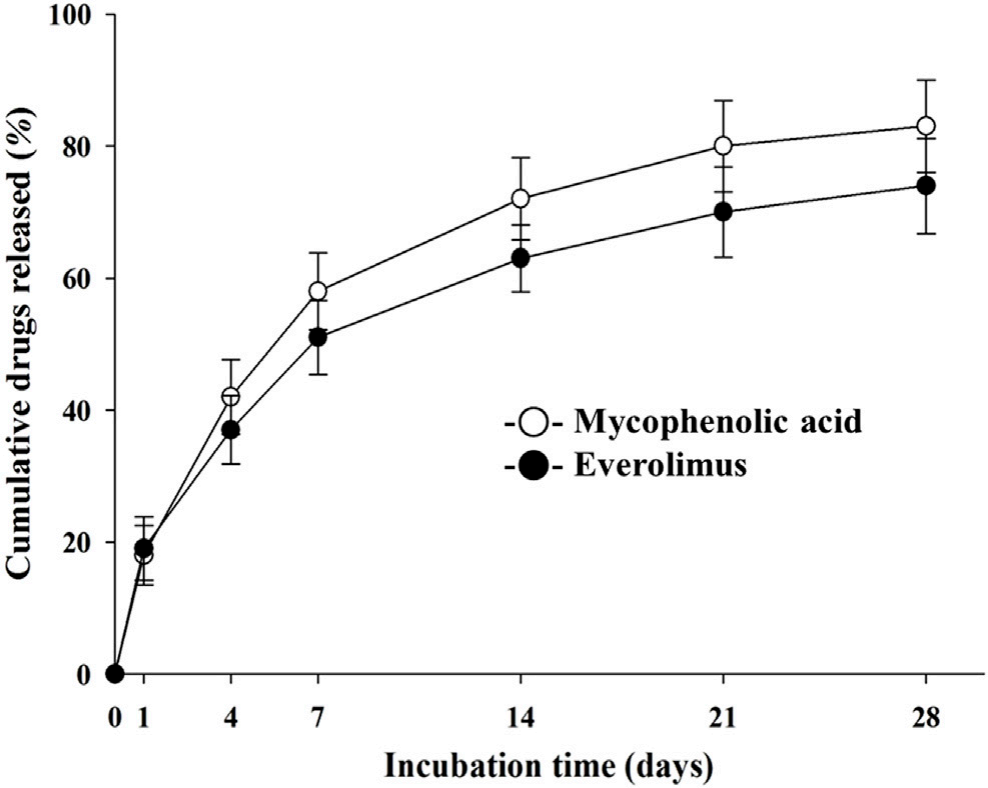
*In vitro* release kinetics of mycophenolic acid and everolimus on the NTM and EES over time. NTM, TiO_2_ film–coated stent with mycophenolic acid; EES, everolimus-eluting stent with biodegradable polymer.

The *in vitro* elution of everolimus or MPA onto the coated stent is shown in Figure 4. The drugs were released continuously over 28 days.

### After Pig Coronary Artery Stenting

Two stents were placed for two coronary arteries in each pig. A total of 30 stents, including 10 NTM, 10 EES, and 10 NT were placed in the middle-proximal left anterior descending and middle-proximal circumflex artery in the 15 pigs. Mortality for this study was zero. There was no significant difference in stent-to-artery ratio among the three stent groups.

### Histologic Analysis Findings Among the Three Groups

**FIGURE 5:**
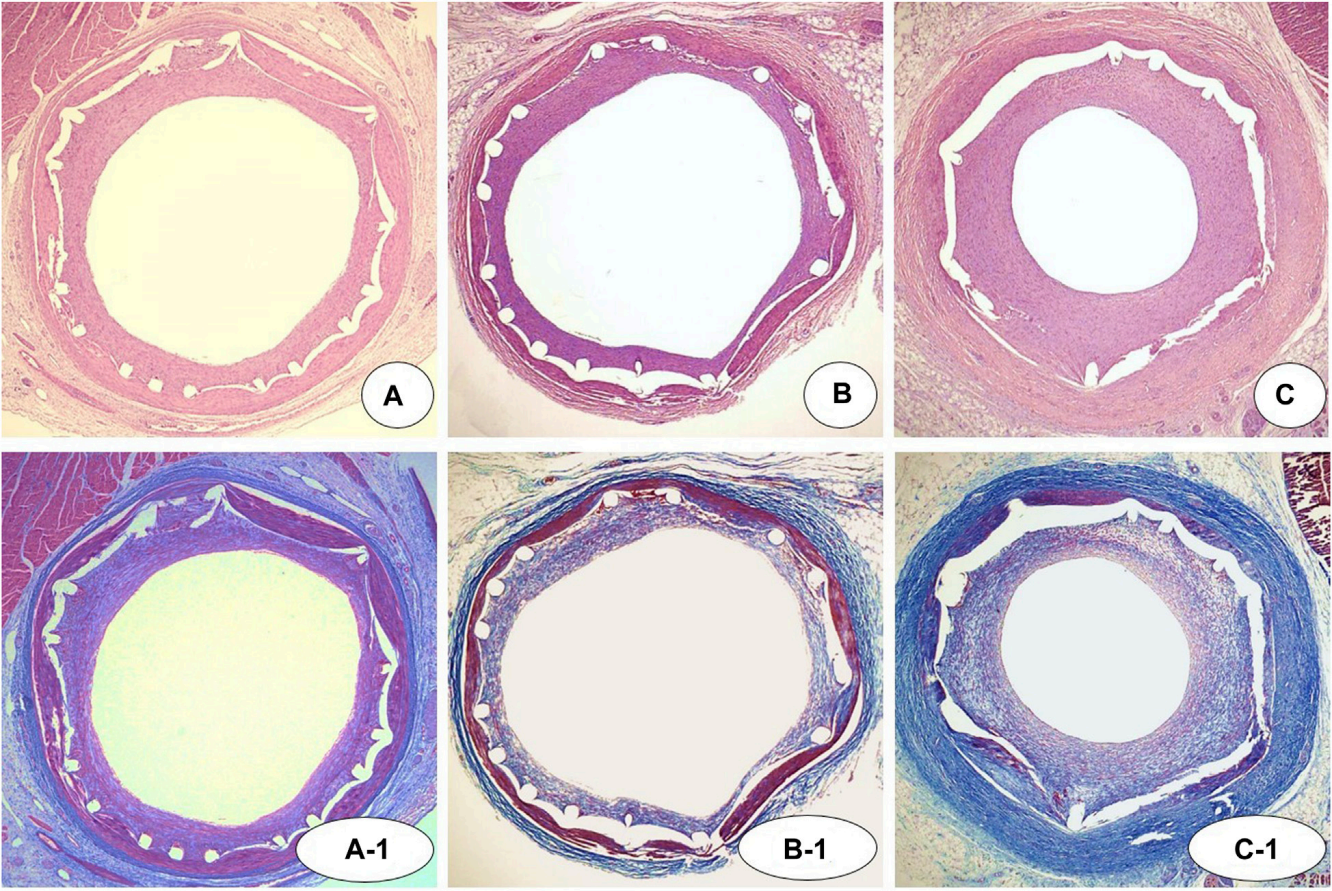
Representative images of hematoxylin-eosin [**(A–C)** ×20) and Carstairs’ fibrin staining (A-1, B-1, and C-1, *×*20) at 4 weeks after stenting. Specimens of implanted NTM [**(A)** and A-1], EES [**(B)** and B-1], and NT [**(C)** and C-1]. NTM, TiO_2_ film–coated stent with mycophenolic acid; EES, everolimus-eluting stent with biodegradable polymer; NT, TiO_2_ film–coated stent.

**FIGURE 6:**
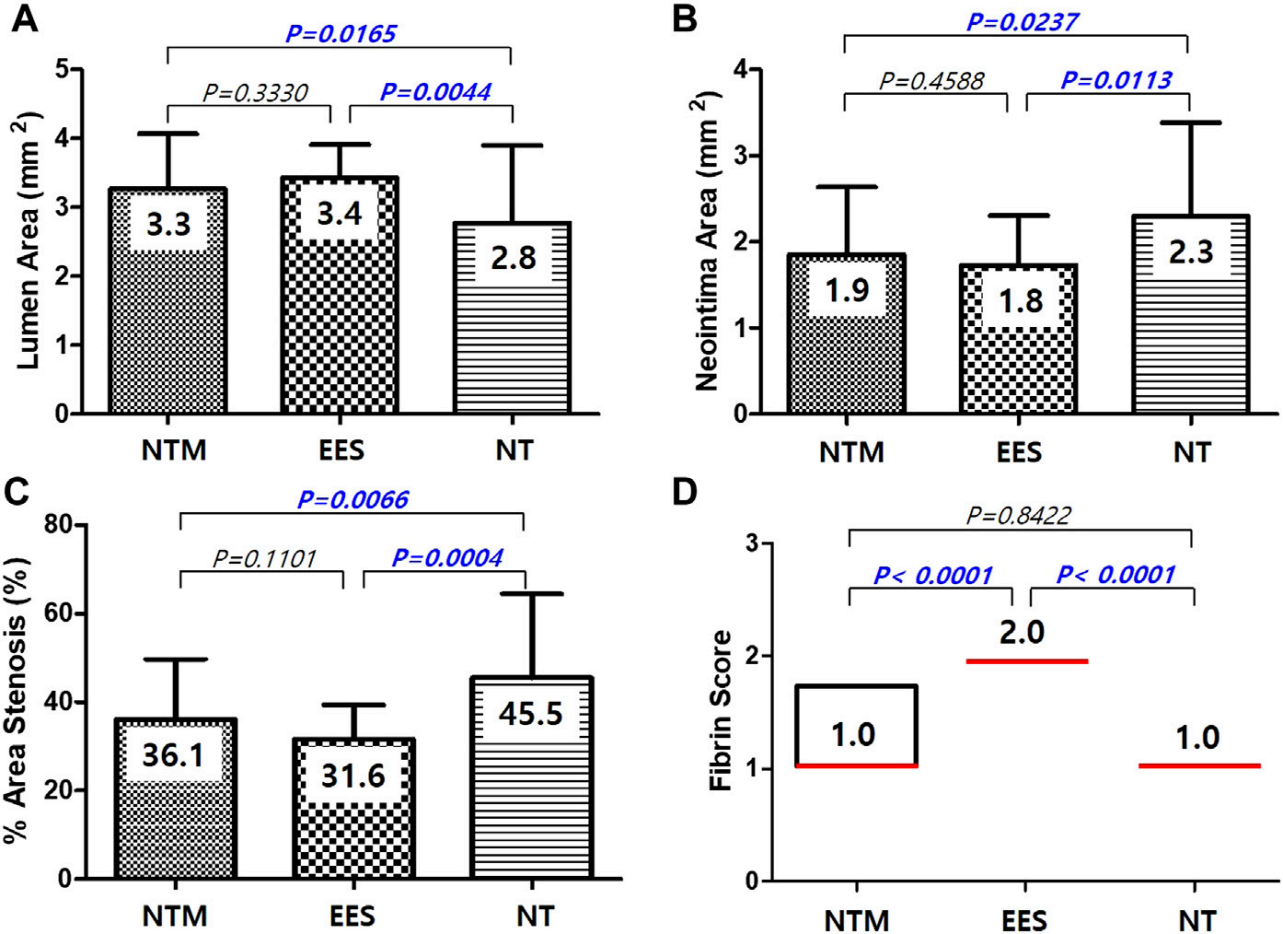
Lumen area **(A)**, neointima area **(B)**, percent area stenosis **(C)**, and fibrin score **(D)** among NTM, EES, and NT. NTM, TiO_2_ film–coated stent with mycophenolic acid; EES, everolimus-eluting stent with biodegradable polymer; NT, TiO_2_ film–coated stent.

There were no significant differences in injury score [NTM, 1.0 (range, 1.0–2.0) vs. EES, 1.0 (range, 1.0–2.0) vs. NT, 1.0 (range, 1.0–2.0); *p* = NS], internal elastic laminar thickness (NTM, 5.1 ± 0.67 mm^2^ vs. EES, 5.2 ± 0.76 mm^2^ vs. NT, 5.1 ± 0.84 mm^2^; *p* = NS), or inflammation score [NTM, 1.0 (range, 1.0–1.0) vs. EES, 1.0 (range, 1.0–1.0) vs. NT, 1.0 (range, 1.0–1.0); *p* = NS] among three groups. There were significant differences in lumen area (NTM, 3.3 ± 0.80 mm^2^ vs. EES, 3.4 ± 0.48 mm^2^ vs. NT, 2.8 ± 1.12 mm^2^; *p* = 0.0038), neointimal area (1.9 ± 0.78 mm^2^ vs. 1.8 ± 0.56 mm^2^ vs. 2.3 ± 1.09 mm^2^, respectively; *p* = 0.0101), percent area stenosis (36.1 ± 13.63% vs. 31.6 ± 7.74% vs. 45.5 ± 18.96%, respectively, *p* = 0.0003), and fibrin score [1.0 (range, 1.0–1.75) vs. 2.0 (range, 2.0–2.0) vs. 1.0 (range, 1.0–1.0), respectively, *p* < 0.0001] among the three groups (Figures 5, 6).

### M-CT Analysis

**FIGURE 7:**
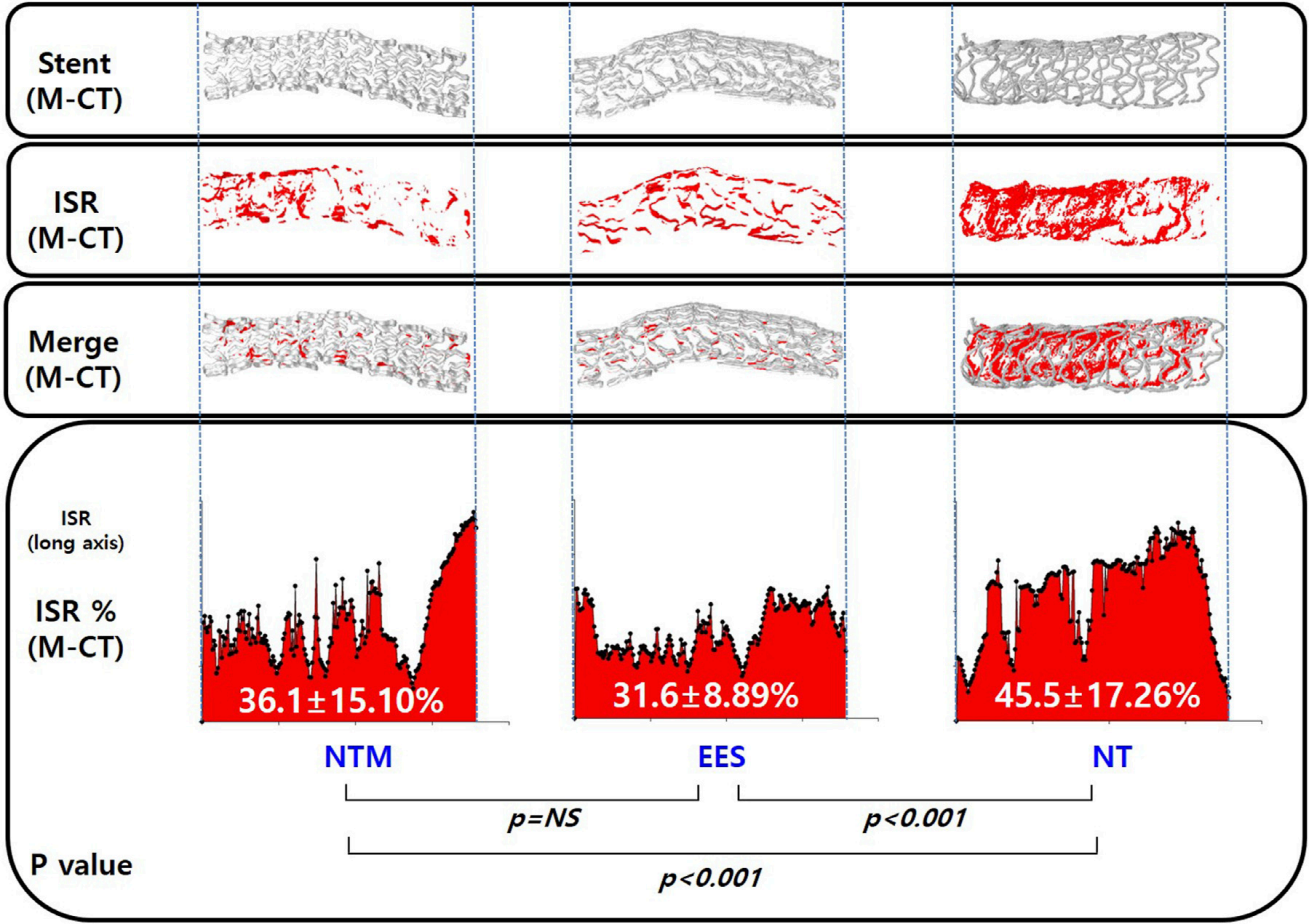
Micro-computed tomography analysis of in-stent restenosis in NTM, EES, and NT. NTM, TiO_2_ film–coated stent with mycophenolic acid; EES, everolimus-eluting stent with biodegradable polymer; NT, TiO_2_ film–coated stent.

The in-stent occlusion rate using M-CT showed similar results to percent area stenosis in the histological analysis (36.1 ± 15.10% in NTM vs. 31.6 ± 8.89% in EES vs. 45.5 ± 17.26% in NT, *p* < 0.05) (Figure 7).

## Discussion

This experiment aimed to compare the NTM (TiO_2_ film–coated stent with MPA) with EES and NT (TiO_2_ film–coated stent) in a porcine coronary restenosis model. Our study demonstrated that NTM had a superior anti-neointimal hyperplasia effect to that of NT and a significantly lower fibrin score than EES.

Currently available metal alloy BMS containing cobalt-chromium, stainless steel, or nitinol can cause vascular inflammation, which leads to a neointimal hyperplasia reaction in stented lesions (Koster et al., 2000). Titanium is commonly used in blood-exposed medical devices such as dental and orthopedic implants because it has superior blood compatibility and stability to those of other metal alloys (Hanawa et al., 1998). When titanium dioxide thin film is coated on BMS, it strengthens the effects of anti-thrombosis, anti-inflammation, and anti-coagulation (Nan et al., 1998).

The final release amount and release rate are summarized in Figure 4. Here, coating with time as a variable revealed a clear trend in the release rate and the cumulative release amount. Since the MPA layer is uniformly coated from the surface of the specimen, the drug release amount is also considered secondarily, to be the basis of controlling the release rate by chemical bonding.(Bozsak et al., 2015; Livingston and Tan, 2019).

A variety of newer-generation DES platforms have been developed to achieve better real-world outcomes such as anti-stent thrombosis and anti-restenosis effects than early-generation products. A recently developed polymer-based DES has shown better clinical results than previous DES platforms, BMS, or balloon angioplasty. However, the underlying problem of the polymer-based DES was the need for longer dual antiplatelet therapy than anticipated. For these reasons, it may not be suitable for use in patients with a high bleeding risk or for whom surgery is scheduled.

The chosen polymer is a very important key factor in drug attachment to the metal stent surface and regulation of drug-release kinetics in DES (Rizas and Mehilli, 2016). Although DES coating techniques using polymer have improved dramatically, permanent or erodible polymers have several side effects. Chronic inflammation is related to polymer triggers such as poor re-endothelialization, delayed arterial healing, and neoatherosclerosis (van der Giessen et al., 1996; Schomig et al., 2007; Otsuka et al., 2014; Otsuka et al., 2015). To solve these problems, we developed polymer-free TiO_2_ thin film–based DES.

In our previous study using non-polymeric DES, TiO_2_ film–coated stent with abciximab (TCA) or alpha lipoic acid (TCALA) showed a superior neointimal inhibitory effect compared to the TiO_2_-coated control stent group, while TCA and TCALA demonstrated significant suppressive effects of inflammation and fibrin deposition compared to commercial biolimus A9-eluting stent using a biodegradable polymer (Lim et al., 2014).

In another previous study, a polymer-free TiO_2_ film–coated stent with everolimus showed superior biocompatibility and compared favorably to a polymer-based everolimus-eluting stent (Sim et al., 2016). As in previous preclinical studies, we established a polymer-free TiO_2_ stent using the PECVD drug manufacturing method.

The most effective drug currently used in coronary stent coating is the immunosuppressive -limus derivatives such as sirolimus (rapamycin), everolimus, zotarolimus, tacrolimus, and biolimus. However, the chemicals in the -limus family do not have carboxyl group. In the manufacture of polymer-free stents, the carboxyl group must be chemically bound to the structure of the -limus family and its yield is very low (less than 10–20% data not shown). Therefore, we have identified MPA as an immunosuppressive agent that satisfies the need for a chemical structure with a carboxyl group.

One way to enhance the interaction of a drug with its biological target is to decrease the conformational flexibility and essentially maintain the drug in its active conformation. A functional group that enhances the water solubility of a drug molecule is often referred to as a hydrophilic functional group. Two main properties that contribute to the solubility of functional groups are their ionization and ability to form hydrogen bonds (Stache et al., 2018).

Therefore, ionization of functional groups formed by plasma modification increases the aqueous solubility of drug molecules and provides the advantage of improved binding interaction with MPA drugs.

Overgrowth of smooth muscle cells after vascular injury play a key role in the restenosis induced by neointimal hyperplasia. MPA inhibited the proliferation of smooth muscle cells induced by endothelin-1, oleic acid, and interleukin-6 inhibition (Fraser-Smith et al., 1995; Mohacsi et al., 1997; Moon et al., 2000; Waller et al., 2005; Ilkay et al., 2006; Ahn et al., 2007; Park et al., 2008; Olejarz et al., 2014).

Although the MPA-eluting stent did not show a treatment benefit in a clinical trial, it is a polymer-based stent that is major different from this study (Carter, 2005).

In the present study using a porcine coronary restenosis model, our novel polymer-free MPA-eluting stent showed higher neointimal inhibitory effect and was not inferior to a polymer-based EES.

In conclusion, here a newer polymer-free titanium dioxide thin film–based DES with MPA showed inhibitory effects of neointimal hyperplasia and delayed arterial healing in a porcine coronary restenosis model.

### Study Limitations

There are two limitations to this study. First, we used normal porcine coronary arteries without atherosclerosis or plaque. Second, follow-up experiments longer than 3 months were not performed.

## Data Availability

The raw data supporting the conclusions of this article will be made available by the authors, without undue reservation.

## Abbreviations

AFM: Atomic force microscopy; AMI: acute myocardial infarction; BMS: Bare metal stentDESDrug-eluting stent; BVS: Bioresorbable vascular scaffold; CNUH: Chonnam national university hospital; Co-Cr: Cobalt chromium; EDS: Energy-dispersive X-ray spectroscopy; EES: Everolimus-eluting stent with biodegradable polymer; ISR: In-stent restenosis; LST: Late stent thrombosis; M-CT: Micro-computed tomography; MPA: Mycophenolic acid; NTM: Titanium dioxide film–coated stent with mycophenolic acid; NT: Titanium dioxide film–coated stent; PECVD: Plasma-enhanced chemical vapor deposition; PTCA: Percutaneous coronary balloon angioplasty; RMS: Root mean square; TiO2: Titanium dioxide.
